# From ArrayExpress to BioStudies

**DOI:** 10.1093/nar/gkaa1062

**Published:** 2020-11-19

**Authors:** Ugis Sarkans, Anja Füllgrabe, Ahmed Ali, Awais Athar, Ehsan Behrangi, Nestor Diaz, Silvie Fexova, Nancy George, Haider Iqbal, Sandeep Kurri, Jhoan Munoz, Juan Rada, Irene Papatheodorou, Alvis Brazma

**Affiliations:** European Molecular Biology Laboratory, European Bioinformatics Institute, EMBL-EBI, Wellcome Trust Genome Campus, Hinxton CB10 1SD, UK; European Molecular Biology Laboratory, European Bioinformatics Institute, EMBL-EBI, Wellcome Trust Genome Campus, Hinxton CB10 1SD, UK; European Molecular Biology Laboratory, European Bioinformatics Institute, EMBL-EBI, Wellcome Trust Genome Campus, Hinxton CB10 1SD, UK; European Molecular Biology Laboratory, European Bioinformatics Institute, EMBL-EBI, Wellcome Trust Genome Campus, Hinxton CB10 1SD, UK; European Molecular Biology Laboratory, European Bioinformatics Institute, EMBL-EBI, Wellcome Trust Genome Campus, Hinxton CB10 1SD, UK; European Molecular Biology Laboratory, European Bioinformatics Institute, EMBL-EBI, Wellcome Trust Genome Campus, Hinxton CB10 1SD, UK; European Molecular Biology Laboratory, European Bioinformatics Institute, EMBL-EBI, Wellcome Trust Genome Campus, Hinxton CB10 1SD, UK; European Molecular Biology Laboratory, European Bioinformatics Institute, EMBL-EBI, Wellcome Trust Genome Campus, Hinxton CB10 1SD, UK; European Molecular Biology Laboratory, European Bioinformatics Institute, EMBL-EBI, Wellcome Trust Genome Campus, Hinxton CB10 1SD, UK; European Molecular Biology Laboratory, European Bioinformatics Institute, EMBL-EBI, Wellcome Trust Genome Campus, Hinxton CB10 1SD, UK; European Molecular Biology Laboratory, European Bioinformatics Institute, EMBL-EBI, Wellcome Trust Genome Campus, Hinxton CB10 1SD, UK; European Molecular Biology Laboratory, European Bioinformatics Institute, EMBL-EBI, Wellcome Trust Genome Campus, Hinxton CB10 1SD, UK; European Molecular Biology Laboratory, European Bioinformatics Institute, EMBL-EBI, Wellcome Trust Genome Campus, Hinxton CB10 1SD, UK; European Molecular Biology Laboratory, European Bioinformatics Institute, EMBL-EBI, Wellcome Trust Genome Campus, Hinxton CB10 1SD, UK

## Abstract

ArrayExpress (https://www.ebi.ac.uk/arrayexpress) is an archive of functional genomics data at EMBL-EBI, established in 2002, initially as an archive for publication-related microarray data and was later extended to accept sequencing-based data. Over the last decade an increasing share of biological experiments involve multiple technologies assaying different biological modalities, such as epigenetics, and RNA and protein expression, and thus the BioStudies database (https://www.ebi.ac.uk/biostudies) was established to deal with such multimodal data. Its central concept is a *study*, which typically is associated with a publication. BioStudies stores metadata describing the study, provides links to the relevant databases, such as European Nucleotide Archive (ENA), as well as hosts the types of data for which specialized databases do not exist. With BioStudies now fully functional, we are able to further harmonize the archival data infrastructure at EMBL-EBI, and ArrayExpress is being migrated to BioStudies. In future, all functional genomics data will be archived at BioStudies. The process will be seamless for the users, who will continue to submit data using the online tool Annotare and will be able to query and download data largely in the same manner as before. Nevertheless, some technical aspects, particularly programmatic access, will change. This update guides the users through these changes.

## INTRODUCTION

ArrayExpress is an archive of functional genomics data, such as gene expression or DNA methylation profiling data (1). ArrayExpress is the main source of data for Expression Atlas (2) – an added-value gene expression database at EMBL-EBI, which allows for gene-, tissue- or disease-based queries. ArrayExpress was established as an archive for microarray data in 2002 (3) as the first MIAME-compliant public database (4). With technology evolving, in 2008 ArrayExpress was extended to accept sequencing-based functional genomics data, in particular, data from RNA sequencing assays (5). For these experiments ArrayExpress stores the processed data and experimental metadata, brokering the sequences to the European Nucleotide Archive (ENA) (6). Starting from 2017, the volume of submissions from sequencing-based experiments has exceeded those from microarrays. For selected transcriptomics experiments, the data are consistently re-processed and re-annotated by our curation team and are made available in Expression Atlas. A major shift over the last two years has been a rapid increase in data from experiments providing cell-level resolution, namely single-cell RNA-seq experiments (7). ArrayExpress is one of the Core Data Resources of the European bioinformatics infrastructure ELIXIR since 2017 (8). Data is submitted to ArrayExpress via the submission tool Annotare (9), which outputs standardized MAGE-TAB format files (10), which are then loaded into ArrayExpress.

Molecular biology experiments are becoming increasingly multimodal and often employ a range of technologies, for example combining RNA-seq, protein expression assays and genotyping. To deal with data from such multimodal experiments, the BioStudies database (www.ebi.ac.uk/biostudies) was established at EMBL-EBI in 2016 (11,12). Its central concept is a *study*, which typically is associated with a publication. BioStudies stores metadata describing the study, provides links to the relevant databases, such as ENA for sequencing or PRIDE for proteomics experiments (13), and also stores the actual data from technologies for which specialized databases do not exist (for instance, microscopy). Thus, BioStudies provides a means to package all the data associated with a (peer-reviewed) publication and provides a more flexible way to organize the data than ArrayExpress. The overarching goal of BioStudies is to support transparency and reproducibility of life sciences research by aggregating all the outputs of a study in a single place. BioStudies also includes the concept of a *collection* of studies, which allows for grouping of datasets that share particular common features or have been acquired from a single source, for example a collection of toxico-genomics datasets gathered in the diXa project (14). An example of linking data from different modalities used in the same study, is study S-BSST390 that contains serial electron microscopy images, links to EMDB entries, a link to the PRIDE database, and a link to another entry within BioStudies.

It should be noted that currently the majority of BioStudies records are created after the related paper has been published and a pipeline from Europe PMC (15) is the main source of data in BioStudies. In fact, a BioStudies record is available for all open articles in Europe PMC that have auto-detected links to life sciences databases, supplementary materials, or both. Other data sources are the SourceData project (16) and image datasets associated with papers in the Journal of Cell Biology. However, we are increasingly focusing on pre-publication data submissions, which allow for citing the respective BioStudies record. Since 2017, BioStudies is an ELIXIR Deposition Database (https://elixir-europe.org/platforms/data/elixir-deposition-databases) and the number of direct submissions is increasing rapidly.

**Figure 1.**
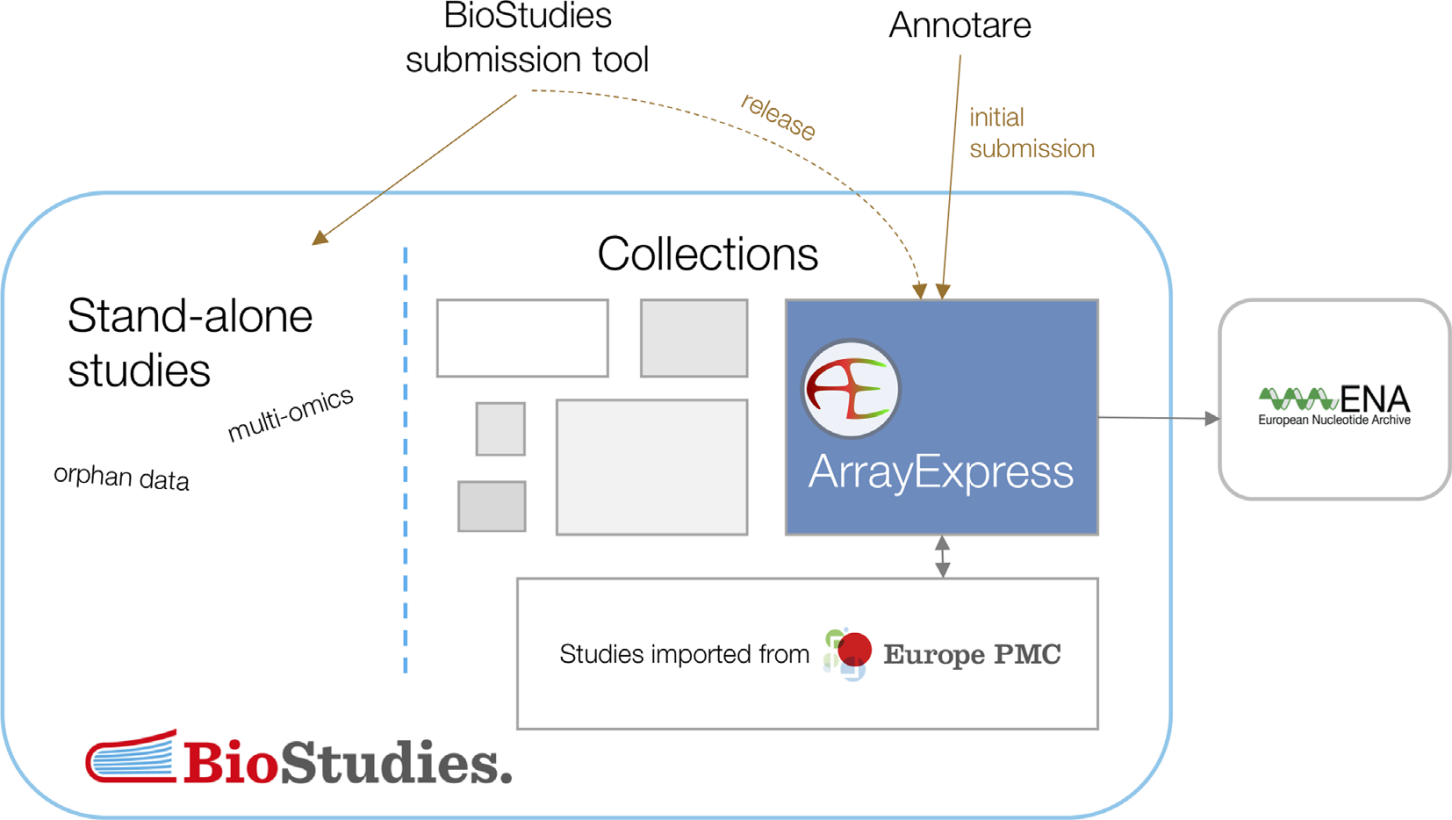
Functional genomics data will be migrated to the ArrayExpress collection in BioStudies and new submissions will be loaded via Annotare into BioStudies (with sequencing data being brokered to ENA).

Being more general and designed with multimodal studies in mind, the BioStudies database will supersede ArrayExpress in 2021. All existing ArrayExpress data will be made available unchanged in BioStudies as a part of the ArrayExpress data collection and all accession numbers will be retained (Figure 1). The BioStudies data search, exploration and API provide all the current ArrayExpress functionality and will be further tuned in response to community feedback. Thus, in the future, all functional genomics data and metadata will be archived in BioStudies. The process will be mostly seamless for the users, who will continue to submit data using Annotare as before and will be able to query and download the data largely in the same manner as currently in ArrayExpress. Nevertheless, some technical aspects, particularly programmatic access, will change, as further detailed below alongside other main developments.

## DATA SUBMISSIONS AND GROWTH

ArrayExpress has continued to grow in data content, and new templates have been added to the Annotare submission tool to improve the user experience, mainly with respect to single-cell RNA-sequencing data.

### Annotare developments

**Figure 2.**
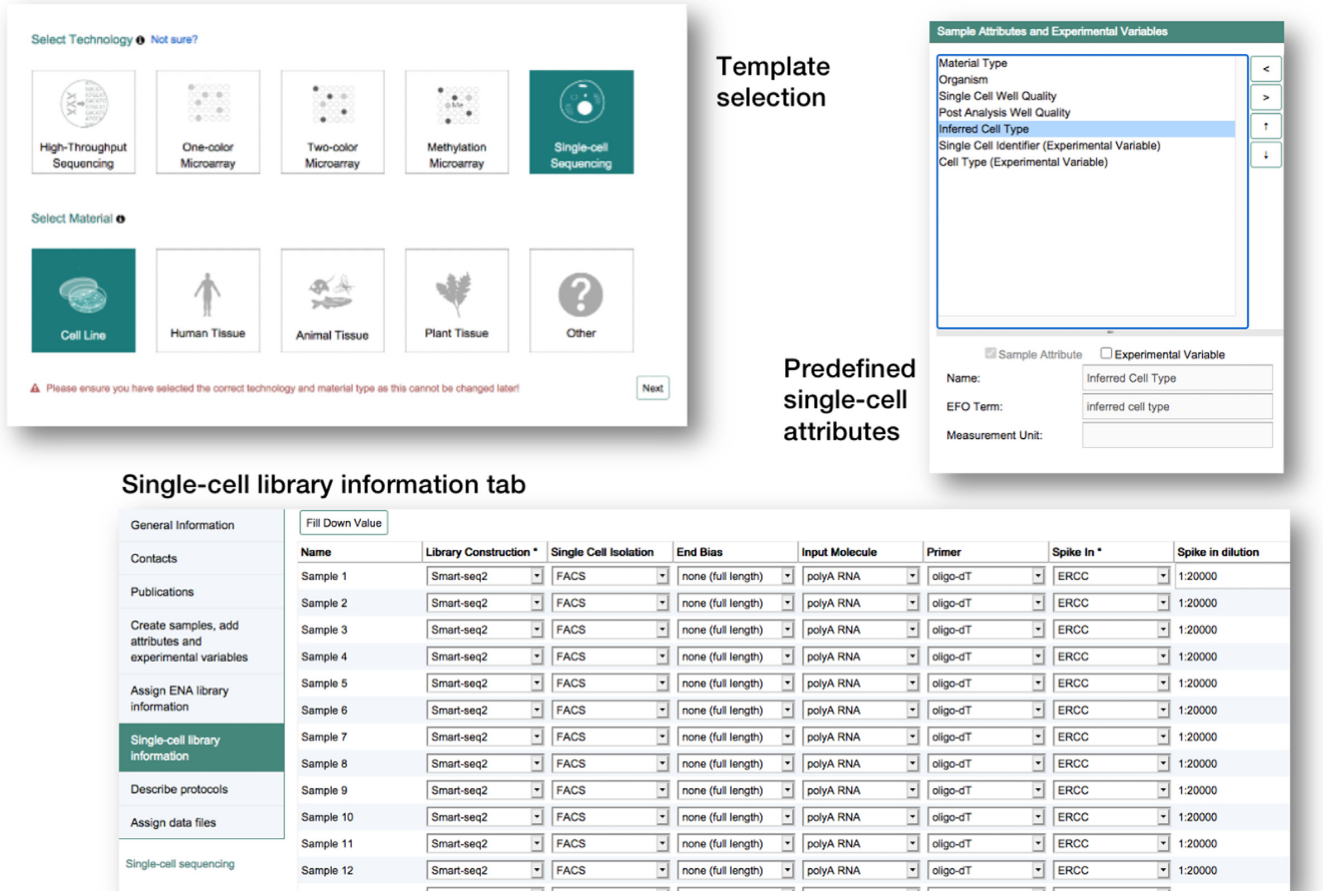
A refined template selection and fields to capture single-cell specific attributes have been added to Annotare.

The single-cell template (Figure 2) provides the submitter with a selection of single-cell specific sample attributes and a new tab to include the details about the single-cell library construction. Here, the user may pick from a list of the most commonly used single-cell protocols, and library attributes, such as ‘end bias’ or ‘UMI barcode size’, will be prefilled with the default values for the selected protocol.

Performance was improved when importing data files after FTP/Aspera upload, and a few changes have improved the user interface, e.g. upload of erroneous file formats is prevented, and useful validation checks and warnings have been added. The submission guide and help content that were previously split between ArrayExpress and Annotare home pages have been integrated into Annotare's website. The submission help has also been updated to the latest EMBL-EBI style framework for a uniform look and feel.

### Submission statistics and trends

**Figure 3.**
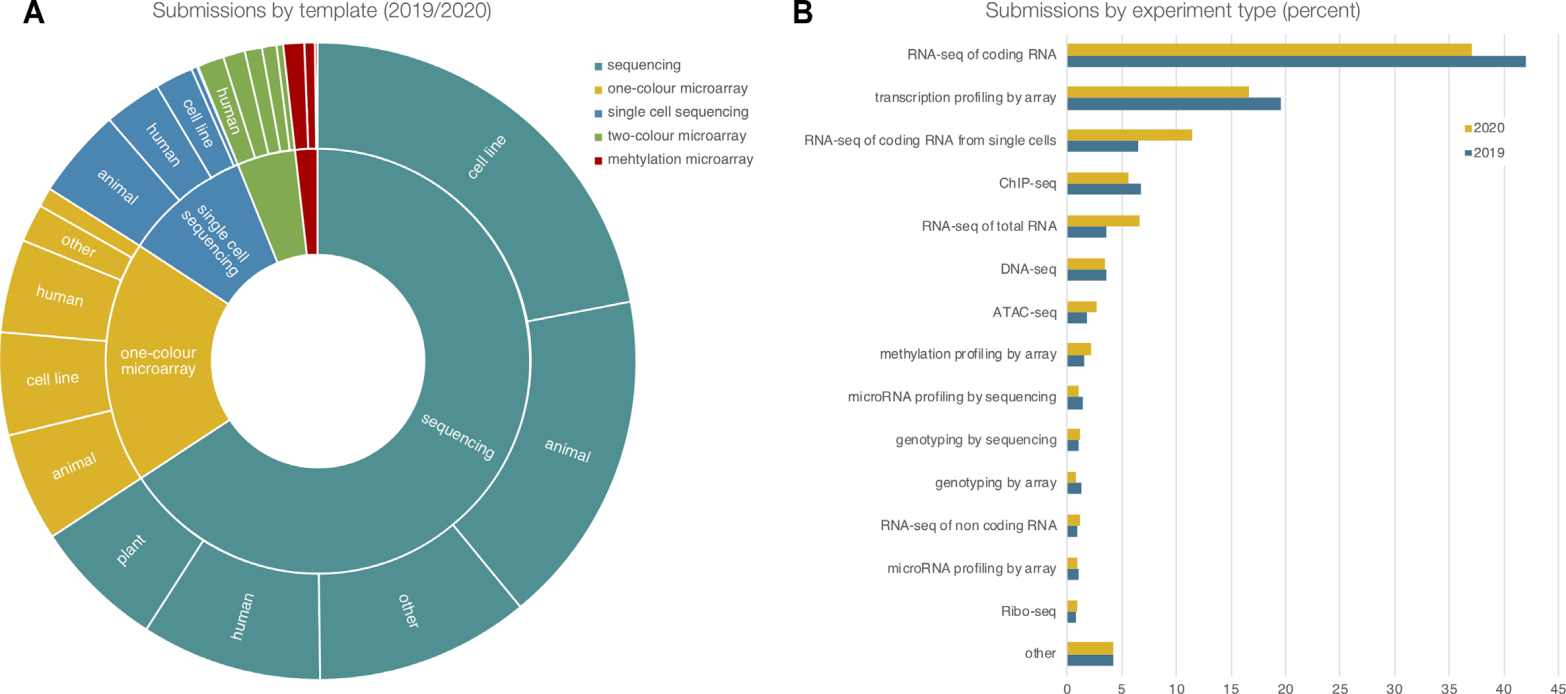
Experiment submissions via Annotare from January 2019 to September 2020; (**A**) broken down by template type, which is composed of technology (inner ring) and biological starting material (outer ring); and (**B**) by experiment type, showing the increase of single-cell RNA-seq (note that ‘RNA-seq of total RNA’ was added new in May 2019).

Since 2017, there have been on average 1000 newly submitted experiments per year. In 2020, single-cell RNA-seq experiments make up more than 10% of the submitted functional genomics experiments while microarray submissions continue to decline (Figure 3). ArrayExpress has recently received its first submission of data produced via the CITE-Seq protocol (17), which generates RNA-seq and protein expression data of cell surface proteins and antibodies in parallel at the single-cell level. Similarly, we have started handling a limited number of spatially resolved transcriptomics protocols, generated in particular via the Visium platform, indicating a trend toward multi-omic and spatial data submissions.

## ACCOMMODATING ARRAYEXPRESS DATA IN BIOSTUDIES

The BioStudies database has been described previously (12), however several new features have been developed, largely to provide the BioStudies ArrayExpress data collection with functionality equivalent to that currently available in ArrayExpress. In particular, our file access interface component can now work with large datasets (millions of files in the same study) and for large data volumes we support FTP and Aspera protocols, both for data depositions and access. A dataset can stay private, i.e. visible only to authorized users, while the associated publication goes through the peer-review process. We also provide a means to filter data via a generic faceting mechanism, which can be easily tuned for the needs of a specific data collection within BioStudies, for easy data exploration. For the ArrayExpress data collection the browser includes facets such as ‘Organism’, ‘Study type’ and ‘Technology type’.

### Data and data representation

While in ArrayExpress the main unit of information is an *experiment*, in BioStudies it is a *study*. These are comparable concepts, and each of the existing ArrayExpress experiments is being translated into a separate study in BioStudies. As already noted, ArrayExpress data entries will retain the accession numbers in BioStudies; moreover, the existing hyperlinks to ArrayExpress will be redirected to BioStudies. All new datasets acquired via Annotare will be loaded into BioStudies, initially in parallel with loading into ArrayExpress, and then exclusively.

The ArrayExpress approach to representing a dataset is based on visualizing the sample-to-data relationship table, which is a part of the MAGE-TAB format (10). BioStudies retains the original MAGE-TAB files and provides a widget for browsing the MAGE-TAB samples table. In addition, utilizing the generic nature of the BioStudies data model we offer other methods to explore a dataset. For example, for each dataset we show a summary table of all unique combinations of experimental factor values, together with the number of samples for these combinations and quick access to the relevant data files. Adding or refining various data representation aspects in response to community feedback is easy to achieve in the BioStudies framework.

### API access

The existing ArrayExpress Application Programmatic Interface (API) functionality will be available from BioStudies, though some changes will happen. API responses in XML format are being deprecated in favor of JSON. However, XML representation is available for individual datasets in a more general schema. We encourage users to shift to the new JSON schema which encapsulates all the information available in the current ArrayExpress JSON. The search API will return a paged response containing only limited metadata about the list of studies being retrieved. Complete metadata on individual studies will not be bundled together, and users will need to iterate through the set and request each study individually. The endpoint changes from https://www.ebi.ac.uk/arrayexpress/xml/v3 to https://www.ebi.ac.uk/biostudies/api/v1. A list of all searchable fields and their BioStudies equivalent is provided in the migration guide available as Supplementary Note and via https://www.ebi.ac.uk/biostudies/ArrayExpress/help. Additionally, the default BioStudies search criteria are available as well.

### Post-submission modifications

ArrayExpress allows specific types of post-submission modifications of the database record to be performed by the submitter, such as changing the public release date or adding a publication reference. Submitting these modifications will be made possible via BioStudies.

### Stages and timeline

The ArrayExpress migration will take place in two phases. During the first phase, scheduled to start in October 2020, all new datasets will be available both from the current ArrayExpress and BioStudies resources. The existing ArrayExpress datasets will become available via BioStudies and the user feedback will be collected. In the second phase, by summer 2021, the current ArrayExpress infrastructure will be deprecated and all data will become a part of the ArrayExpress data collection in BioStudies.

## FUTURE PLANS

The ArrayExpress to BioStudies process is still on-going. For instance, currently Annotare and BioStudies have separate user accounts for user authorization. This will be addressed by supporting the ELIXIR AAI system (18), which will allow for a single login to all EMBL-EBI data resources. EMBL-EBI maintains a range of databases for specific types of life science data and knowledge (19), most with custom-built data deposition. The submissions of multimodal experiments to the EMBL-EBI resources is a more general question, that goes beyond the remit of this ArrayExpress update and here we discuss the relevant future developments only briefly.

The BioStudies future developments directly relevant to ArrayExpress data submitters are related to Annotare. The development of Annotare will focus on improvements for more efficient submission throughput and handling of larger submissions. As both the number of submissions and their respective size increase (in terms of sample numbers and data volume), faster file import, validation and submission processing will be developed. Changes on the interface will be implemented for easier annotation of large numbers of samples. We will also continue to adjust the templates to the possible shifts in technologies. As techniques such as CITE-Seq and spatially resolved transcriptomics become more commonly used, we will expect to make updates in the MAGE-TAB representation of metadata for these types of studies.

For well-established types of data, such as gene expression or protein mass spectrometry, a custom-built data deposition tool offers the best support for their users and ensures the reusability of the collected data. Typical examples of such tools are Annotare and the PRIDE proteomics data submission tool (13). However, a more generic data acquisition system is needed for data generated by new technologies during the early stages of their development, e.g. currently for light microscopy (20). To serve both needs, BioStudies offers two submission systems: Annotare for gene expression and functional genomics data collected to the standard that enables population of Expression Atlas, and a more generic data submission tool for types of data for which specialized resources do not yet exist. Unfortunately, this means that currently to submit data from multimodal experiments to the EMBL-EBI resources, several separate submissions may be needed, which then can be linked via BioStudies (the linking happens automatically for data related to publications available in Europe PMC). The roadmap toward simplifying this process will be described in the next BioStudies update. The migration of ArrayExpress to BioStudies is part of our work to simplify EMBL-EBI’s data resource ecosystem.

This is the last ArrayExpress update in the Nucleic Acid Research Journal Database Issue since future updates will be a part of the BioStudies database and Expression Atlas updates. However, we would like to emphasize that all the data submitted to ArrayExpress since 2002 will be available from BioStudies database without any changes and the accession numbers will be preserved. The ArrayExpress migration to BioStudies reflects the changing field of life sciences, where multimodal high-throughput experiments have become a norm.

## Supplementary Material

gkaa1062_Supplemental_File
