# Supplementary material for: From ArrayExpress to BioStudies
Source: Nucleic Acids Res. 2020 Nov 19;49(D1):D1502–6. doi: 10.1093/nar/gkaa1062 (PMC7778911; doi:10.1093/nar/gkaa1062)
Supplement: gkaa1062_Supplemental_File [file gkaa1062_supplemental_file.pdf]

ArrayExpress to BioStudies API Migration Guide

We are in the process of migrating ArrayExpress to BioStudies as a collection. As a result, there would be some changes in the API support for ArrayExpress. Until announced, the current version of the API will continue to work. Some of the major changes are:

XML to JSON

XML API responses are being deprecated in favour of JSON. However, XML representation is available for individual experiments in a more general PageTab schema. We encourage you to shift to the new JSON schema which should have all the information available in the current AE JSON.

Response bundling

Search API will return a paged response containing only the metadata about the experiments. Results would not be bundled and you'll have to iterate through the set and request each experiment individually.

Endpoint changes

The endpoint has changed from `https://www.ebi.ac.uk/arrayexpress/xml/v3` to `https://www.ebi.ac.uk/biostudies/api/v1`. A list of all searchable fields and their BioStudies equivalent is given below. These fields are in addition to the default BioStudies search criteria fields. Please send all questions and feedback to biostudies@ebi.ac.uk.

Current Field	New Field	What is searched?	ArrayExpress Example	BioStudies URL
accession		Experiment primary ArrayExpress or secondary (GEO, ENA, EGA etc) accession	`<endpoint>/experiments?accession=E-MEXP-31`	`<endpoint>/studies/E-MEXP-31`
array	TBD	Array design accession or name (wildcards supported)	`<endpoint>/files?array=A-AFFY-33`	`<endpoint>/files/A-AFFY-33`
expdesign	experimental_design	Experiment design type, related to the questions being addressed by the study, e.g. "time series design", "stimulus or stress design", "genetic modification design". Has EFO expansion.	`<endpoint>/files?expdesign=dose+response`	`<endpoint>/search?experimental_design="dose response"`
exptype	study_type	Experiment type, related to the assay technology used. List of experiment types in ArrayExpress. Has EFO expansion.	`<endpoint>/experiments?exptype="RNA-seq of non coding RNA"`	`<endpoint>/search?study_type="RNA-seq of non coding RNA"`
ef/ev	experimental_factor	Experimental factor (also called experimental variable), the name of the main variable under study in an experiment. E.g. if the factor is "sex" in a human study, the researchers would be comparing between male and female samples, and "sex" is not merely an attribute the samples happen to have. Has EFO expansion.	`<endpoint>/experiments?ef="cell type"`	`<endpoint>/search?experimental_factor="cell type"`

efv/evv	experimental_factor_value	The value of an experimental factor. E.g. The values for "genotype" factor can be "wild type genotype", "p53-/-". Has EFO expansion.	<endpoint>/experiments?efv=HeLa	<endpoint>/search?experimental_factor_value=HeLa
sa	source_characteristics	Sample attribute values, e.g. "male", "liver". Has EFO expansion.	<endpoint>/files?sa=fibroblast	<endpoint>/search?source_characteristics=fibroblast
sac	source_characteristics_value	Sample attribute category that is defined in an experiment, e.g. "age", "cell type", "disease". Has EFO expansion.	<endpoint>/files?sac=age	<endpoint>/search?source_characteristics_value=age
species	organism	Species of the samples. Can use common name (e.g. "mouse") or binomial nomenclature/Latin names (e.g. "Mus musculus"). Has EFO expansion.	<endpoint>/experiments?species="homo sapiens"	<endpoint>/search?organism="homo sapien"
pmid		PubMed identifier	<endpoint>/experiments?pmid=16553887	<endpoint>/search?link_type=pmid&link_value=16553887
gxa	gxa	Presence ("true") / absence ("false") of an ArrayExpress experiment in the Expression Atlas.	<endpoint>/experiments?gxa=true	<endpoint>/search?gxa=true
directsub		If "true" only returns experiments directly submitted to ArrayExpress (i.e. not imported from GEO). For more information about how we import data from GEO see the GEO data help page.	<endpoint>/experiments?directsub=true	TBD
raw	raw	Experiment has raw data available.	<endpoint>/experiments?raw=true	<endpoint>/search?raw=true
processed	processed	Experiment has processed data available.	<endpoint>/experiments?processed=true	<endpoint>/search?processed=true
assaycount	assay_count	The number of of assays where x <= y and both values are between 0 and 99,999 (inclusive). To count excluding the values given use curly brackets e.g. assaycount={1 TO 5} will find experiments with 2-4 assays. Single numbers may also be given e.g. assaycount=10 will find experiments with 10 assays.	<endpoint>/experiments?assaycount=[1 TO 5]	<endpoint>/search?assay_count=[1 TO 5]
samplecount	sample_count	The number of samples	<endpoint>/experiments?samplecount=[1 TO 5]	<endpoint>/search?sample_count=[1 TO 5]
efcount	experimental_factor_count	The number of experimental factors	<endpoint>//experiments?efcount=[1 TO 5]	<endpoint>/search?experimental_factor_count=[1 TO 5]
